# Professional development arising from multiple-site workplace learning: boundary crossing between the education and clinical contexts

**DOI:** 10.1186/s12909-020-02225-y

**Published:** 2020-09-23

**Authors:** Margot Barry, Wietske Kuijer-Siebelink, Loek A. F. M. Niewenhuis, Nynke Scherpbier

**Affiliations:** 1 Academy of Allied Health Professions, HAN University of Applied Sciences, Kapittelweg 33, 6525EN Nijmegen, the Netherlands; 2 Interprofessional and Collaborative Practice in Health Hand Social Care, HAN University of Applied Sciences, Nijmegen, the Netherlands; 3 Faculty of Education, HAN University of Applied Sciences, Nijmegen, the Netherlands; 4 Open University, Welten-Institute for Research on Learning, Teaching and Technology, Heerlen, the Netherlands; 5 Primary Care Specialty Training Department, Department of Primary and Community Care, Radboud University Medical Centre, Nijmegen, the Netherlands

**Keywords:** Boundary crossing, Broker-role, Professional development, Workplace learning

## Abstract

**Background:**

This research explores the value of an inter-organisational jurisdiction, on the professional development of faculty members in their roles of researcher and educator. Faculty members from a Dutch university of applied sciences, who work in both the education and clinical practice contexts, participated in this research.

**Methods:**

Individual semi-structured interview were conducted with nine faculty members, from various academic health professions, on their experiences of professional development arising from working in both the clinical and education contexts. In this exploratory, post-positive interview study, interview transcripts were analysed thematically.

**Results:**

Participants reported that working in two organisational contexts, whilst performing two faculty roles that span both contexts, enhances their ability to broker connections between research, teaching and practice. The boundary crossing activities which participants performed, contributed to professional development in all faculty roles. The broker role was not seen as being a unique role which is distinct from research and practice roles. Broker activities were seen as generic and supportive of the roles that bestow academic status and expertise.

**Conclusions:**

To the participants in this research, the relevance of the broker role in professional development is not as evident as the relevance of roles that enhance specialisation and subject specific expertise. They consider broker activities as supportive to the roles of researcher and teacher. The broker role is time consuming, but not yet visible as a distinct professionalisable work-package in its own right. It is also not well defined in literature. A better understanding of the broker role could lead to its development in order to harness its professional development potential tenably across academic roles.

## Background

Faculty members in academic health professions are known to fulfil a variety of roles simultaneously, including those of educator, researcher and expert in their own field of practice [1]. Those performing multiple roles have the ability to act as brokers [2], in facilitating connections within the so-called “research-teaching-practice triangle” [3]. Meaningful connections between research, teaching and practice contribute to a sustainable curriculum in applied teaching [4]. This is of particular interest to universities of applied science, which are the focus of this research, where links between the academic- and practice environments are considered strategically important. Connections between teaching and practice, can contribute to the delivery of practice-ready graduates to employers [5]. Connections between research and practice can yield clinically useful research results [6]; and connections between research and teaching can facilitate improved undergraduate teaching [5].

Competing responsibilities of multiple roles are known to be professionally challenging [7], and faculty members with such roles need to create a “work-work balance” in the performance of tasks across different roles [8]. Additionally, faculty members need to maintain currency in multiple domains. These domains include teaching and learning, research, and their own specific fields of expertise or practice [1]. Their professional development is therefore multi-faceted by virtue of the multiple roles they perform.

Faculty roles in academic health professions are operationalised in two main organisational contexts: the education setting (university of applied science) and the clinical practice environment. Faculty members operate in one or both of these [1]. The clinical practice context and the education context are known to differ in culture and organisational logic [9]. The differences between these contexts can cause boundaries, which are socio-cultural differences that cause discontinuity and difficulties in interaction between the two contexts; these boundaries are multi-faceted in nature [9, 10]. An example of such a boundary, is the situation, in which clinical professionals perceive large proportions of students as being ill-prepared for their practice placements. The students are unable to participate effectively in the clinical environment. Clinical professionals might assume that the education environment did not fully comprehend the needs of the clinical environment and failed to prepare students effectively. Conversely, education professionals might assume that clinical professionals lack skills in nurturing student learning. Such boundaries can be lowered through various means including brokers who work in both organisational contexts to facilitate improved interaction and coordination between these [9].

The theory on learning through boundary crossing [10] introduces the notion that fulfilling a broker role, which concerns itself with lowering of boundaries between related yet disparate contexts, could contribute to professional development.

Maintaining related yet diverse faculty roles whilst brokering a connection between two organisational contexts simultaneously, could therefore have a positive effect on professional development within the research-teaching-practice triangle. This research seeks to explore, how those working in both the education- and clinical context view the effect of their boundary spanning jurisdiction across two organisational contexts, on their professional development in multiple faculty roles.

This research attempts to understand the value of the inter-organisational broker role in the professional development of the faculty roles of educator and researcher. Before describing the methods, we present a summary of relevant literature:

### Professional development of faculty members

Faculty development programs support the professional development of academic staff by introducing social structures, which in and of themselves do not automatically lead to professional development. They provide learning opportunities in situated work processes, which an individual can choose to interact with in order to achieve professional development [11]. Professional development activities for faculty members encompass diverse formal and informal learning activities [1]. Formal professional development activities such as courses, conferences and e-modules, are easily understood, visible, quantifiable and justifiable [12]. Informal professional development activities are not easily quantifiable and often not visible, though they play a vital role in gaining tacit knowledge and becoming a more competent professional [1, 12]. Many informal learning activities are ubiquitous to everyday work [13]. Workplace learning as an approach to professional development has received very little attention in research publications in health professions education and this has been identified as a research gap [1]. This research wishes to contribute to the body of knowledge on workplace learning as an approach to faculty development by exploring the effect of working in two related contexts on the development of faculty roles. The theory of learning through boundary crossing was used to examine professional development across boundaries of organisational contexts.

### Boundary crossing

Attempts to link related yet disparate contexts creates potential for professional development through boundary crossing [6, 10, 14]. However, not every difference between contexts is a boundary, only those that lead to difficulties in constructive cooperation. Boundaries between contexts with divergent goals and disparate organisational logics will always exist, and it is not realistic to aim at eradicating these. However, boundaries can be lowered or crossed in meaningful ways to facilitate cooperation between contexts [9]. Boundary crossing is described as the attempt which is made to improve or restore continuity of action and interaction between contexts [15]. Wenger [16] described three manners in which boundaries can be crossed: firstly, boundary interactions: communication and collaboration between people across boundaries; secondly, boundary workers or brokers: people that work across boundaries for example dual-role professionals and thirdly, boundary objects: artefacts that are used in different contexts and have a function in each context.

The relationship of this theory to professional development lies in the assertion that boundaries hold learning potential [10]. Constructive engagement with boundaries leads to professional development through the following four learning mechanisms: identification, reflection, coordination and transformation [10]. There is an individual and organisational dimension in this type of professional development where the individual as part of an activity system at work always has an effect on the context and vice versa [17]. Boundary crossing can occur at intra-personal, inter-personal and organisation levels [18]. Therefore, whilst this research attempts to look at the perspective of the individual faculty member, their learning and development is always to be seen as situated within their work context.

Insights gained might assist faculty developers to structure workplaces in a manner that they become pedagogically rich professional development opportunities for faculty members.

### The broker-role

Professionals who perform a broker role, engage in activities that connect two related yet disparate context with each other such as for example clinical practice and the education context. Broker activities can be performed intra- or inter organisationally to expand networks and to drive change [19]. Connecting the roles of researcher and educator within an educational institution is an example of intra-organisational brokering whilst connecting a clinical practice context with a university is an example of inter-organisational brokering. Broker activities are distinct from research, practice and teaching activities [20]. In health professions three categories of broker activity have been identified in a systematic review as being implemented to connect disparate contexts and roles. These broker activity categories are networking ('linkage'), capacity building and knowledge management [2]. Some difficulties have been associated with broker role performance in academic health professions. Firstly, it is often assumed that a connection is being brokered by a person who has professional jurisdiction in two contexts or roles. The exact nature of the broker role at the operational level is not explicit in literature and the exact nature of broker activities is unclear [6]. Brokers can therefore occupy an ambiguous space between two roles which causes them to experience professional identity difficulties [21]. The identity formation of dual-role professionals in academic health professions has received attention in research literature [14, 22], however, the development of the broker role at the operational level less so [6, 23]. Secondly, in an age of specialisation, broker activities such as networking and capacity building could be seen as being fairly generic and of low value when compared with tasks that relate to subject specific expertise [20]. Faculty members in a boundary spanning situation might therefore tend towards the development of their subject specific expertise at the expense of investing in the ill-defined work package that is the broker role.

## Context

The “Link-Structure” is a faculty development programme offered at a Dutch university of applied science. In the Netherlands, universities of applied science have recently transitioned from being educational institutions with a more vocational focus (called “Hogeschool”), to having university status and a more academic focus. The universities of applied science intend to retain strong links with practice whilst strengthening the research role within their institution [22].

The Link-Structure allows lecturing staff to work in a clinical practice setting for one or two days per week to perform embedded research, and if desired clinical work, as part of their faculty role. Faculty members are linked with a clinician and expected to conduct a “substantial research project” on a research question they identify with the clinician in the clinical practice setting. The faculty member remains employed by the university and the clinician remains employed by the clinical practice setting. The clinician works for an equivalent period of time in the higher education setting to compensate the absence of the lecturer. Where this is logistically difficult PhD funding becomes a manner of compensating for the absence of the faculty member from teaching tasks.

The nature of the work activities which faculty members perform (research or clinical) in the clinical setting are not stipulated by the university: they are negotiated between the faculty member and the clinician(s) involved. The university describes this as embedded research that links education, research and clinical practice through personnel resources. Embedded research implies a state of “in-between-ness” and calls for broker activities such as relationship building, knowledge generation in co-creation and capacity building [24].

The research question for this research is: *What do individual faculty members report about the effect that working in the organisational contexts of higher education and clinical practice, has on their professional development in the faculty roles of teaching and research?*

## Methods

We chose a qualitative, explorative research methodology to answer the research question [25]. We engaged in purposive sampling, which is a non-probability sampling method based on participants’ characteristics and the purpose of the study. We invited lecturers who worked both in the educational- and clinical settings whilst simultaneously holding the faculty roles of educator and researcher. We sent a letter of invitation and information by email to all lecturers who participated in the Link-Structure at that time as they fulfilled these criteria (*n* = 11). The clinicians participating in the Link-Structure, whilst fulfilling part-time educational tasks, were not invited to participate as they did not meet all the criteria. Lecturers were invited to participate in individual, semi-structured interviews on a voluntary basis, to talk about their professional development arising from working in both the education and clinical contexts, whilst holding faculty roles of educator and researcher. The interviews were conducted by the lead author (MB) at the university where the participants work. Each interview was audio recorded with the consent of the participant. The five key interview questions, with their associated follow-up questions from the interview guide were as follows:

1. What motivated you to participate in the Link-Structure?
2. How do you experience working in two contexts, the education and clinical contexts? (Follow-up question: can you give concrete examples of your experiences?)
3. How would you describe the opportunities, which the Link-Structure offers you to progress your professional development as an educator? (Follow up question: What do you perceive as facilitative and hindering conditions in the reported opportunities?)
4. How would you describe the opportunities, which the Link-Structure offers you to progress your professional development as a researcher? (Follow up question: What do you perceive as facilitative and hindering conditions in the reported opportunities?)
5. Could you give some concrete examples of your work activities in the Link-Structure?

The interview guide was not piloted but discussed extensively with co-authors NS and WK.

An independent observer (LD), who has a PhD in ethics, attended the interviews and acted as a critical friend [25] during the data analysis stage. LD’s independent perspective was important given that MB worked as a lecturer (MSc) and PhD-candidate, at the same university as the participants. MB did not participate in the Link-Structure but knew four of the participants from day-to day work at the university.

Interviews were transcribed verbatim. We conducted a thematic analysis in Microsoft Word which followed a process of reading, re-reading, inductive coding, categorising and deriving themes [26]. MB and LD coded the first two interviews together and MB coded the remainder of the interviews. The co-authors (WK, LN, NS) discussed the process of categorising and formulating themes to increase the quality of the analysis [25].

In our post-positive approach to analysis [27], sensitising concepts were those of boundary crossing [10], brokerage [20] and workplace learning [28]. Therefore, whilst we coded inductively, we expected to find information linked to these concepts.

The interviews were conducted in Dutch. We translated in-vivo quotes from participants that were used for this article into English. All participants were given the opportunity to read the translation of their own words in the context of this article to determine whether the English translation captured the meaning and emotion they intended to convey. One participant suggested an amendment to one translation and this was made. Reading the translations in the context of this article was also used as an opportunity for member checking [25]. In accordance with the information from the ethics advisory board (Ethies Advies Commissie FGGM), this research did not require ethical testing under the Dutch Law “Wet medisch-wetenschappelijk onderzoek met mensen (WMO)” [29] as it was conducted with consent-competent adults and carried no known risks to participants. We observed the national guidelines for good research conduct at university level [30] and all participants signed consent forms.

## Results

Nine out of the eleven faculty members in the Link-Structure participated in individual interviews which were between 40 and 60 min long. Two declined participation on account of limited time. Among the three male and six female participants the disciplines of physiotherapy, occupational therapy, speech and language therapy, dietetics and health technology were represented. The clinical practice settings they worked in were tertiary hospitals and rehabilitation facilities. The participants ranged in age from 32 years to 47 years. Each participant worked in professional practice for at least 5 years before assuming their faculty position in higher education. The length of time spent working in higher education ranged from 4 to 11 years. The length of time of participation in the Link-Structure ranged from 8 months to 4 years. Research interests of the group included topics such as mobility difficulties, scleroderma, chronic fatigue, computer assisted articulation, and use of technology in health care provision. More detailed participant information cannot be given to safeguard confidentiality.

The thematic analysis resulted in six themes that elucidate the nature of professional development arising from participation in the Link-Structure as experienced by the participants. After seven interviews, no new codes were added indicating that data saturation was reached for this group.

### Theme 1: individual motivation to approach professional development opportunities

Participants reported that they chose to participate in the Link Structure because of their interest in re-establishing links with healthcare practice in which they perceived themselves to possess expertise. They reported positive affective associations with practice. Participants also reported ambitions to progress professionally through research.

*“I just found it nice to go back into practice”* and *“update* (my) *knowledge of healthcare practice”*. *“I had built up a certain level of expertise in the past on the topic of [____] and I felt that I wanted to contribute to practice through my expertise.”* (R4). *“I just wanted to observe what happens in practice to go along and see what they do and what they talk about.”* (R7) *“I had the ambition to do a PhD. The expectation in the Link Structure is to do a PhD. Therefore, I started it (participation in the Link Structure)”* (R3).

The participants’ words indicate that they are motivated by the performance of tasks that relate to subject matter expertise and professional development as a researcher. Their primary motivation for engagement did not to originate in wanting to consciously broker a connection between research, practice and teaching.

### Theme 2: professional identity across contexts

Participants described themselves as having multiple professional identities.

*“I see myself primarily as a researcher and thereafter as a teacher. But to be able to do both of these jobs well I need to be excellent at my field of expertise. I go into practice to keep up my professional practice knowledge.”* (R8) *“When I am here at the university, I am one of the teachers but when I am in the practice setting, I am one of them -therapist. So, when I talk about “us” it is a different “us” in practice than here.”* (R3).

A number of identities known in literature for research-active academics at universities of applied science [22], were discernible. The participants described themselves as researcher, disciplinary expert and evidence-based teacher. None of them, however, reported having an identity that related to being a broker for example the identity of ‘liaison officer’. They associated different identities with different context and appeared to separate these from each other. Their engagement in the clinical practice setting informed their roles of educator and researcher but they did not report seeing themselves as someone who connected the two organisational contexts with each other.

### Theme 3: intra-personal boundary crossing as a mechanism for professional development

Participants identified differences between the education context and the clinical context which existed in the domains of culture, organizational structure, language use and conceptualization of certain theoretic constructs. Observing these differences encouraged them to learn. Learning mechanisms associated with boundary crossing [10], identification and reflection, were identifiable in their words. Identification and reflection on differences in culture between contexts led to intra-personal learning through boundary crossing.

*“My tunnel vision decreased … . It (the hospital) is a totally different place of work but also a different culture. This is within the discipline of occupational therapy and also the academic teaching hospital at large. It has a very different work culture than we have in university and that is interesting. It provides me with a lot of food for thought to be able to move within it, to learn … because I often make mistakes”* (R3) *“Actually I have to say that I saw things in practice which I had read about in a book but to see them in practice is totally different. Also, the people (are different). Yes, the healthcare language, healthcare practice is a totally different world … it was an eye opener”* (R7).

Participants described how their engagement with information from one setting transformed their work in the other. The use of information from the clinical setting not only enriched the content of their lessons but also contributed to their credibility as perceived by the students.

*“I can give a very nice example, whereby I was in the ICU one day with a lady who was knocked off her bike by a motorist. The next day I had a case example for a head injury during a lecture. That really fitted very well. These are the cases that you now use in your lectures.”* (R1) *“ … .obtaining and using real-life case examples of patients during lessons with students … this makes my lessons more interesting but the students also view me in a totally different light”* (R3).

Through the engagement across contexts, the participants started identifying and reflecting on differences which increased their understanding of the clinical context in relation to the education context. They also started using first-hand experiences from clinical practice as boundary objects in their teaching, thereby facilitating practice-teaching connections. In these ways, they perceived their teacher role to improve and develop through their engagement in clinical practice.

### Theme 4: research as a boundary activity

Despite not seeing themselves as a broker at the outset of their engagement in the Link-Structure, participants reported that their research linked the clinical context with the education environment in various individually mediated meaningful ways. Research projects and the researcher’s role were described as a link between education and clinical practice. Participants increasingly presented research results gained in the clinical setting during lectures in the education setting. Elements of PhD research formed the basis for bachelor students’ theses and faculty members felt that doing research themselves increased their competence in supervising student research. Participants reported that they learnt a lot from their PhD trajectory in the link-structure. The following words of participants illustrate these findings:

“*That is the crazy thing about research: the link with practice* *and* *the link with education and I really see it and I am enthusiastic about it.* M*y research skills really have a bearing on my daily student education activities.” (R5)*.

*“A lot (of my work in the Link Structure) relates to my PhD and therefore also a lot to students bachelor theses … . I have presented the results that arise (from my PhD research) in one of the elective modules for undergraduates. Therefore, what is found (in research) ultimately lands again in student education.”* (R1).

I had to *“supervise student research but the fact that I did research myself was a factor in doing this effectively … I could caution students about things that I had done wrong previously … In any event, the research that I do. I learn a lot from that.”* (R6)

The boundary activity between research- in- practice and teaching, developed as participants progressed their embedded research and continuously engaged with knowledge and work processes in the clinical context and education context simultaneously. A familiarity of both contexts allowed them to use the role of researcher to link the contexts through knowledge management and capacity building.

### Theme 5: networking and relationship building as part of the learning process

The theme networking encapsulates the participants’ description of important social processes that are necessary to access learning opportunities across contexts. The outcomes of building effective professional networks and behaviours are described. Network behaviours such as being habitually and predictably present, being visible and being reciprocal in work activities were reported as important. The establishment of a network in the clinical setting benefited the faculty members themselves who got to work with experts. It also benefited their students who had more learning opportunities and better support during practice placements in the faculty members professional network.

“ *… networking is very important and at the beginning I did not realise how important it is … Networking is about things like this, being present at lunch (*in the hospital) *every Monday*” (R3), “*being predictably present in the clinical setting at the same time every week*” (R6), and “*setting clear rules of engagement with each other with regards to attendance*” (R1).

“*I work on … things that are important for the hospital but that are also important for my PhD. So, in this manner we have made a connection that works for both*. *… If a course is to be held, I can secure (free) facilities for this. It is nice that the people from the hospital conduct their courses here in the university building. I mean it gives us exposure and the prestigious logo (of the hospital) adorns the course” (R1). “We can see what can be done together … . in collaboration. It seems good to look out for each other.”* (R7)

“*I spend time with our own students here on placement. It is difficult for a practitioner in the paediatrics department who does not work at the university to understand what students know, how they are being educated and what they could mean in your department … what kind of expertise they might have that could be meaningful. And, if you work in the university you might not understand the kind of expertise necessary to work in the paediatrics department … . you cannot simply place a student and expect them to find their feet themselves. No, it (the practice placement) could not have worked without my presence here*.” (R2)

Of all the participants, only R8 reported an awareness and an attempt to contribute to his team of colleagues in the education setting. *“From time to time I discuss my Link Structure activities with my head of department to see how our education setting can also benefit from my participation in the Link Structure”* (R8).

Networking (linkage) is known as an important activity category in brokering connections between contexts. However, participants did not report planning strategic networking activities. They noticed during their performance of Link-Structure related work, how important networking behaviour was as a pre-requisite for engagement across contexts for themselves and their students. Networking facilitated personal links and collaboration opportunities in the research-education-practice triangle.

### Theme 6: Organisational facilitators for professional development

In addition to the professional development processes mentioned in the five themes above, several practical and administrative elements associated with the Link Structure were seen as pre-requisites for professional development. These were, the association between the university and the clinical context at institutional level, the protected time, the freedom of choice in the research topic and the high degree of autonomy in designing of own work engagement in the clinical setting.

*“It is helpful to be taken off the roster (for teaching tasks) for a little while. Then you really have the freedom to think. I have freedom and that motivates me … ”* (R7). “T*he exact tasks to be conducted in the practice setting were not dictated … . the freedom of choice and the luxury of time played the most important role in the ability to plan and work on projects”* (R3).

Having legitimate access to a second work context without requiring a second employer was seen as a facilitating factor to participating in professional development opportunities offered in the Link Structure.

*“I have previously had multiple employers and I don’t find that pleasant. Of course, now I have more people I have to take into account but with regards to financing and administration everything is clear.”* (R2)

In faculty development and workplace learning opportunities, the practical pre-requisites play an important part in whether or not the learning opportunity is tenable for an individual. In the case of the Link-Structure it appears that the pre-requisite conditions not only facilitated but also motivated engagement.

## Discussion

For the participants in this research, the ability to work in two related organisational contexts (clinical practice and education) whilst performing their academic roles of researcher and teacher contributed to their professional development in various ways. At the outset, participants were motivated to improve their research and practice skills through habitual access to the clinical practice context. They engaged in tasks that were congruent with their professional identities of researcher, teacher and subject matter expert. In their work processes, the participants described boundary crossing activities and opportunities to broker a connection between contexts and roles, as part of their professional development. They engaged in networking, capacity building and knowledge management to connect research, teaching and practice. At the outset, the participants had not anticipated or planned these broker activities pertinently as a means of professional development. Yet, the broker activities constituted a large portion of the activities they described as part of their professional development. The facilitative practical conditions supported professional development across contexts.

The results agree with existing research [1, 11], in that both the individual’s disposition and organisation’s conditions play a role in the professional development of faculty members. Their habitual presence in the clinical context contributed to expertise in clinical practice whilst the roles of researcher and educator spanned the boundaries of the education and clinical contexts. The presence of the two organisational contexts in addition to two boundary spanning faculty roles, secured representation of work activities on all three points on the research-teaching-practice triangle.

It is notable that the participants did not plan the execution of broker activities to develop in their role as researcher and teacher. The brokering opportunities seemed to arise as they engaged across contexts, and they learnt from participation in work processes. Given that brokerage facilitates learning through boundary crossing [18], the broker activities contributed to their professional development, whether they were conscious of this or not.

Despite limited descriptions of the broker role in literature [23] the participants in this research were able to give extensive descriptions of their broker activities. They did not, however, consider their broker role as distinct from their role of researcher and teacher, and this could contribute to an underestimation of the time-investment necessary for broker activities. In many publications the broker role is also not pertinently considered as a separate role [6]. Connections are assumed to occur where professionals work in multiple contexts. In the description of the Link-Structure the broker role is also not pertinently mentioned, whilst the expectation of linking research, practice and teaching is explicated. It is possible that the broker activities (networking (linkage), knowledge management and capacity building [2]), are perceived as generic skills which do not contribute to specialisation or subject matter expertise. And in the tradition of academic health professions, subject matter expertise and specialisation are highly valued in the context of professional development [20, 23]. Broker activities could therefore be seen as activities that are supportive of roles that bestow professional status, whilst they constitute an entire work package in its own right [20]. Attempting to perform a broker role on the periphery, as a subset of other roles, might intensify a number of occupational disadvantages associated with the performance of multiple roles. These disadvantages include work overload, time pressure, having to contend with competing interests of different roles, professional identity difficulties and a feeling of not belonging entirely to one distinct community of professionals [22]. In addition to these disadvantages, the performance of the broker role leads to CPD outcomes that are in most instances not easily quantifiable and usable for regulatory CPD requirement. The burden of these difficulties could lead to a reduced appreciation and limited investment in broker activities, especially in the absence of an understanding of the distinctiveness of the broker role.

Increased awareness of the uniqueness and value of the broker role in its own right could benefit its development and its contribution to the professional development of faculty members performing multiple roles across multiple contexts.

### Limitations of this research

A limitation of this research is the limited number of participants that all came from the same higher education institution, resulting in a strong selection bias. The findings are not generalisable to a wider population and they apply to this specific group of respondents. The findings are, however, of interest to a wider population of faculty members. This research explored the views of individual faculty members who chose to participated in the Link-Structure and were consequently positive about working in two contexts. The research does not provide insight into a broader organisational perspective or views of managers and colleagues with less interest in forging links between research, practice and teaching. These limitations provide a starting point for further research, which could focus on the nature of the broker role longitudinally and the organisational impact of individuals’ broker activities. In addition, a larger sample of faculty members from a wider array of higher education institutions could be recruited.

## Conclusion

This research shows that working in two organisational contexts enhances faculty members’ ability to broker connections between research, practice and teaching through their faculty roles that span the education and clinical practice contexts. Concrete activities are executed to broker connections in the research-teaching-practice triangle. The broker role might appear generic but makes concrete contributions to professional development through boundary crossing. A better understanding of the broker role could lead to better support and tenable development of this role and its professional development potential.

## Data Availability

The datasets (in the Dutch language) used during the current study are available from the corresponding author on reasonable request.
